# Cytotoxicity and Antitumor Activity of Arglabin and its Derivatives

**DOI:** 10.3889/oamjms.2023.11114

**Published:** 2023-02-24

**Authors:** Sergazy Adekenov, Vojtech Spiwok, John Beutler, Olga Maslova, Kairolla Rakhimov

**Affiliations:** 1 JSC, International Research and Production Holding “Phytochemistry”, Karaganda, Republic of Kazakhstan;; 2 Department of Biochemistry and Microbiology, University of Chemistry and Technology, Prague, Czech Republic;; 3 Molecular Targets Laboratory, Center for Cancer Research, National Cancer Institute, Bethesda, Maryland, USA

**Keywords:** Sesquiterpene lactones, Arglabin, Molecular docking, Cytotoxicity, Antitumor activity, Farnesyl protein transferase, Topoisomerases -I and -II

## Abstract

**BACKGROUND:**

At present, more than 8000 sesquiterpene lactones have been isolated and described from natural sources, a significant part of which has cytotoxicity and antitumor activity. One of the practically available sesquiterpene lactones is arglabin, which, as a renewable material, is used for the synthesis of new compounds. The article presents data on the study of cytotoxicity and antitumor activity of the arglabin and its derivatives using molecular modeling methods and, in the experiment *in vitro* and *in vivo*.

**AIM:**

The aim of this work is to study the cytotoxicity and antitumor activity of new compounds based on the sesquiterpene lactone arglabin using molecular modeling and experimental pharmacology.

**METHODS:**

ChemDraw programs and a set of AutoDock programs were used for computer simulation. Molecular docking was carried out using the Maestro graphical interface of the Schrödinger Suite software package (Schrödinger, LLC, New York, NY, 2017). Docking modes standard precision and XP (extra precision) were used. In *in vitro* experiments, the antitumor activity of compound samples was studied in models of 60 human tumor cell lines, and clonogenic C6 rat glioma cells. The antitumor activity of the samples was studied in experiments *in vivo* on white outbred rats with transplanted tumors and was evaluated by the inhibition of tumor growth and the magnitude of the increase in average life expectancy.

**CONCLUSION:**

When studying the antitumor activity on 60 cell lines of tumor cells (NCI60), clonogenic cells of C6 rat glioma, a high antitumor activity of some arglabin derivatives was established. The connection between the structure of arglabin derivatives and their inhibitory effect on farnesyl protein transferase, topoisomerases -I and -II was studied.

## Introduction

In recent years, effective cytostatics based on natural compounds have been increasingly used in modern medicine. High expectations are placed on natural compounds of plants, the action of which is aimed at new cellular targets for solving one of the main problems of modern clinical oncology-multidrug resistance of tumors. A targeted search for new cytostatic agents involves, first of all, the development of molecules that can actively influence the pathological cells of the body and their growth.

One of the promising classes of natural compounds in this field is sesquiterpene lactones, many of which are cytotoxic *in vitro*, and some demonstrate an antitumor effect *in vivo* [1].

This is a large group of natural terpenoids with a wide range of biological activities: anti-inflammatory, cytotoxic, cardiotonic, analgesic, antispasmodic, hypoglycemic, hypotensive, antibacterial, antifungal, etc. [2]. To date, the structures of over 6000 sesquiterpene lactones have been established, and the largest number of them has been isolated from flowering plants - representatives of *the Asteraceae (Compositae)* family.

Cytotoxic sesquiterpene lactones include costunolide 1, eupatolid 2, parthenolide 3, dehydrocostuslactone 4, thapsigargin 5, and arglabin 6 [3]. On the basis of the sesquiterpene lactone derivative arglabin, JSC “IRPH “Phytochemistry” developed the antitumor drug “Arglabin” [4].

Sesquiterpene α-methylene-γ-lactones exhibit cytotoxicity, antiviral, antimicrobial, and antitumor properties. One of the important features of sesquiterpene lactones is the presence in their structure of an α-methylene-γ-lactone fragment, which is responsible for biological activity, especially antitumor activity. An exocyclic double bond conjugated to a carbonyl function is an alkylating agent and can act on transcription factors and enzymes in the human body. And it is assumed that the exomethylene group conjugated to the carbonyl group in the lactone ring is responsible for the cytotoxicity of sesquiterpene lactones [5–7].

The aim of this research is to study the cytotoxicity and antitumor activity of new derivatives of the sesquiterpene lactone arglabin using computer modeling and experimental pharmacology methods.

## Materials and Methods

The following derivatives of the sesquiterpene lactone arglabin were presented for the study: α-epoxyarglabin 7, β-epoxyarglabin 8, dioxyarglabin 9, dimethylaminodioxyarglabin 10, and dimethylaminodioxyarglabin hydrochloride 11.

*In vitro* antitumor activity of the compounds was studied on a model of 60 human cell lines of tumor origin. At the first stage of screening, three highly sensitive human cell lines MCF-7 (breast carcinoma), NCI-H460 (lung carcinoma), and SF-268 (glioma) were supplemented with the test substance at a standard concentration and incubated for 48 h. NCI60 is based on the SRB method for determining the viability of cell cultures using the pink anionic dye sulforodamine B [8]. If the test substance inhibits the growth of at least one cell line, it proceeds to the next stage of testing on a full panel of 60 cell lines. The test substance was then added to the cells at five different concentrations.

Furthermore, *in vitro* antitumor activity was studied on the model of clonogenic C6 rat glioma cells, i.e. experimental *in vitro* model of human glioblastoma multiforme, rat C6 glioma cell line 8–14 passages *in vitro*) and on the primary culture of *in vitro* cells isolated from biopsy material of children with brain gliomas (medulloblastoma) [9], [10].

*In vivo* antitumor activity of the samples was studied on white outbred rats with transplanted tumors of mice and rats. The antitumor effect of the studied compounds was determined by daily intraperitoneal injection in a 2% solution of dimethyl sulfoxide (DMSO) for 5 days at the maximum tolerated dose [11], [12]. To evaluate the antitumor activity of the compounds, we used the percentage inhibition of tumor growth and the value of the increase in life expectancy (IILE), determined immediately after the end of treatment.

Cytotoxicity was studied under *in vitro* conditions in the survival test of larvae of marine crustaceans *Artemia salina (Leach)* (Brine shrimp toxicity bioassay method) [13], [14]. The analysis was carried out according to the method proposed by Meyer and McLaughlin with minor modification.

The test was carried out using readymade samples at a concentration of 100 μg/mL, 10 μg/mL and 1 μg/mL, as well as positive and negative controls, which were 13-dimethylamino-1,1β-epoxy-5,7α,6 hydrochloride, 11β(Н)-guai-3,4-en-6,12-olide (substance of the Arglabin drug), which has antitumor (cytotoxic) activity, and DMSO, used to dilute the test compounds, in three parallel experiments. Each test sample and control sample were transferred to different labeled tubes into which about 10 actively swimming shrimp were released. The tubes were stored at room temperature at about 22°C. Lethality was observed within its range of activity and was assessed after 24 h of exposure. The analysis was carried out in triplicate and the values were recorded in vials containing 5 ml of solution and 10 shrimp. To determine IC_50_ values, eight different concentrations were tested [15].

Three-dimensional structures of receptors were taken from the RCSB PDB database (http://www.rcsb.org/): Human DNA topoisomerase I - PDB ID: 1SEU; human DNA topoisomerase II - PDB ID: 3QX3 and human farnesyl transferase - PDB ID: 1S63 which are intracellular targets of cytotoxic and antitumor drugs [16], [17].

Molecular docking was carried out using the Glide program of the Schrödinger Suite (Schrödinger, LLC, New York, NY, 2017). Docking modes standard precision and XP (extra precision) were used.

As the final results, the value of the scoring function *G-score* was used, showing the energy and strength of binding of the ligand to the target molecule.

### Statistical analysis

Statistical processing of the results was carried out using “The GraphPad Prism v. 6.0.” The obtained results are presented as “mean value ± standard error of mean value.” Differences were considered significant at the achievement significance level p<0.05 using the Kruskal–Wallis H test.

## Results and Discussion

### Molecular docking of arglabin and its derivatives

According to the results of the docking, it was found that the presented molecules of sesquiterpene lactones showed interaction with the receptors of DNA topoisomerase I, DNA topoisomerase II, and farnesyl transferase (Table 1).

Dioxyarglabin 9 and dimethylaminodioxyarglabin hydrochloride 11 showed relatively high binding energies to DNA topoisomerase II (−9.0 and −9.0 kcal/mol, respectively) and to DNA topoisomerase I (−8.4 and −7.1 kcal/mol, respectively).

α-Epoxyarglabin 7, β-epoxyarglabin 8, and dimethylaminodioxyarglabin 10 showed relatively strong binding to DNA topoisomerase II (−7.8, −7.6, and −7.6 kcal/mol, respectively) and to DNA topoisomerase I (−7.0, −6.0, and −6.8 kcal/mol, respectively).

Dioxyarglabin 9, dimethylaminodioxyarglabin 10, and dimethylaminodioxyarglabin hydrochloride 11 showed relatively high binding energies to farnesyl transferase (−7.4, −7.3, and −7.0 kcal/mol, respectively). A relatively strong bond with farnesyl transferase was shown by α-epoxyarglabin 7 and β-epoxyarglabin 8 (−6.6 and −5.5 kcal/mol, respectively).


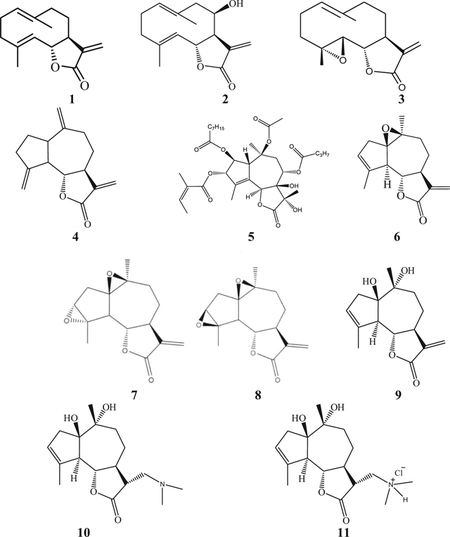


Dimethylaminodioxyarglabin hydrochloride 11 showed the best values of binding energy with all three studied biological targets for cytotoxic activity: DNA topoisomerase I, DNA topoisomerase II, and farnesyltransferase (−−8.4, −9.0, and −7.5 kcal/mol, respectively).

**Figure 1:**
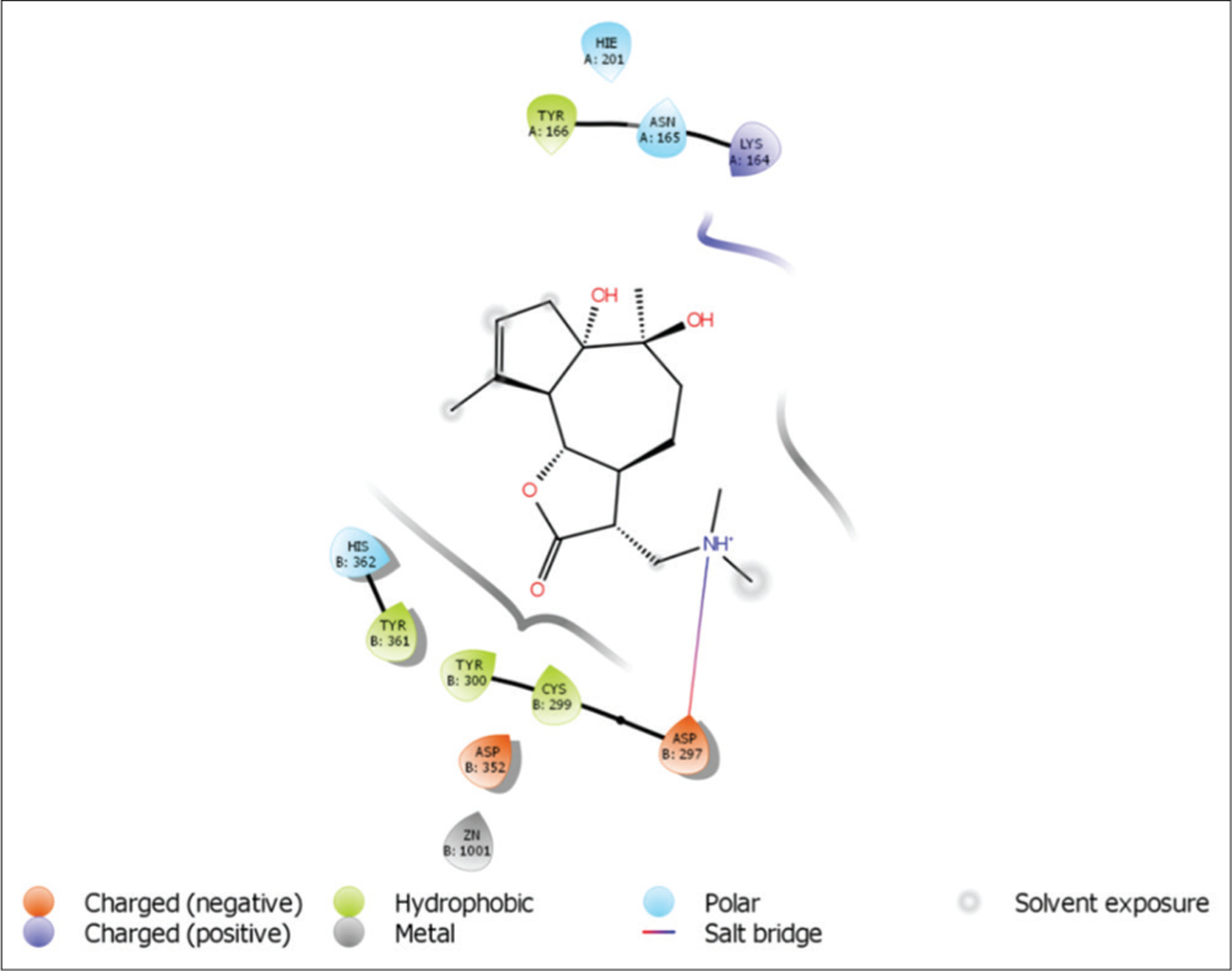
Interaction of farnesyl transferase with dimethylaminodioxyarglabin hydrochloride 11

**Figure 2:**
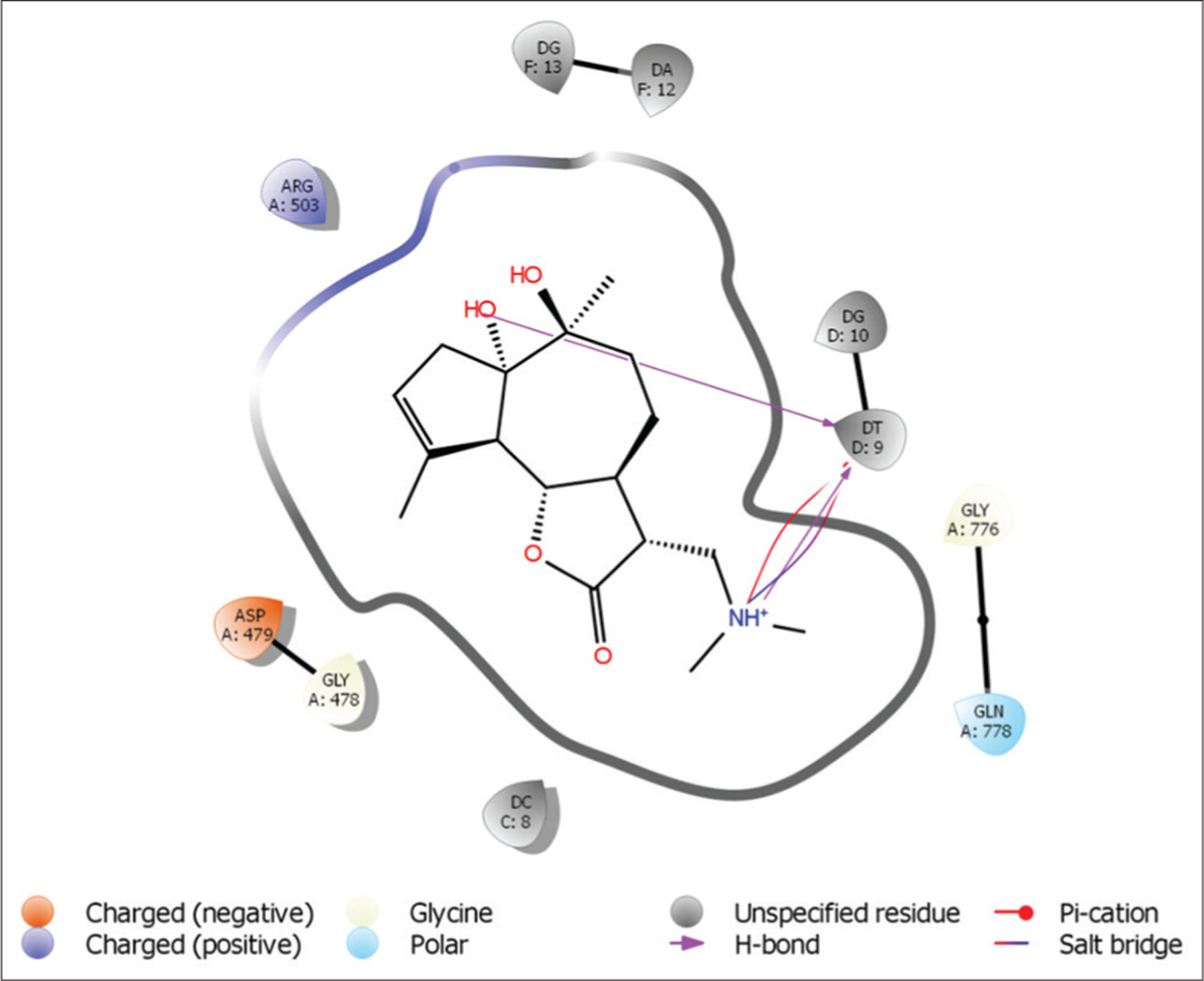
Interaction of DNA topoisomerase II with dimethylaminodioxyarglabin hydrochloride 11

**Figure 3:**
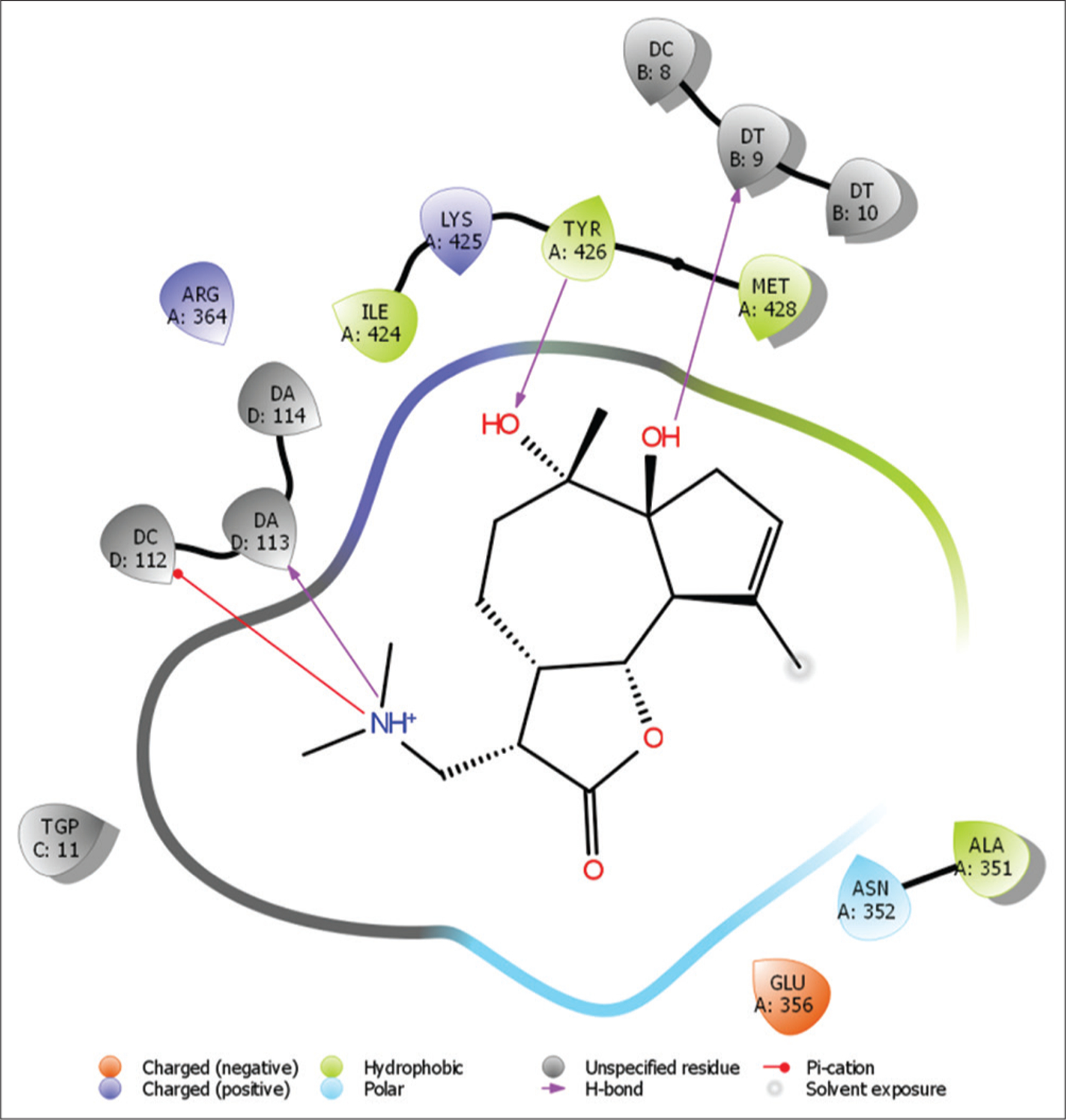
Interaction of DNA topoisomerase I with dimethylaminodioxyarglabin hydrochloride 11

The obtained high binding energies of the studied arglabin derivatives with biological intracellular anticancer drug targets and DNA-processing enzymes suggest a mechanism of their cytotoxic action, which prevents the repair of breaks and causes the accumulation of damaged DNA molecules, thereby forcing the death of the tumor cell (Figures 1–3).

### Antitumor activity on rats with transplanted tumors

Antitumor activity in relation to transplanted strains indicates the prospects of searching among them for new agents for the chemotherapy of malignant tumors (Table 2).

It was found that the presence in the sesquiterpene lactone molecule of such alkylating centers as α-methylene-γ-lactone, α, β-unsaturated keto group, epoxy cycle, as well as functional groups such as the hydroxyl function, halogen atoms contribute to the inhibition of the growth of tumor strains [18].

In the series of sesquiterpene lactones with a guayanic structure, arglabin 6 and its modified derivatives exhibited high antitumor activity.

The study of the antitumor activity of arglabin 6 and its derivatives characterizes the different effect of their action on strains of 8 transplanted tumors [19]. Considering the dependence of the antitumor activity of arglabin 6 and its derivatives on the structural features of their molecules, the following should be noted:

- The introduction of halogen atoms (bromine and chlorine) enhances the antitumor effect, so 3,4-dibromide arglabin 15 inhibits the growth of strains of Pliss lymphosarcoma (LPS), alveolar liver cancer PG-I, sarcoma M-I and sarcoma-45 by 70–90%, and dichlorodihydroxyarglabin 16 inhibits the growth of sarcoma-45, sarcoma M-I and LSP resistant to prospidin, up to 71%.
- Epoxidation of arglabin at the C3-C4 skeletal double bond also increases its antitumor activity. Thus, β-epoxyarglabin 8 in MPD (30 mg/kg) significantly inhibits the growth of sarcoma-45, sarcoma M-I (78–88%), alveolar liver cancer PC-I, Pliss LPS, and breast cancer (59–88%). 72%. This compound 8 in MPD has a pronounced antitumor activity against Pliss LPS, resistant to leukoephdin (80% inhibition), rubomycin (78.0%) and sarcoma-45, resistant to 5-fluorouracil, prospidin and rubomycin (66.0–78%) [20].
- Among the amino derivatives of arglabin, dimethylaminoarglabin 17 and its hydrochloride 18 show pronounced antitumor activity.

Of interest is dimethylaminoarglabin hydrochloride 18, highly soluble in water, which turned out to be practically important in the preparation of a rational prescription for the dosage form of the drug.

Dimethylaminoarglabin hydrochloride 18, when intraperitoneal and intratumor injection, inhibits the growth of LL lung carcinoma, Ehrlich solid tumor, Ca-755 breast adenocarcinoma, and sarcoma 37 by 86–90%; lymphocytic leukemia P-388 (IILE-84%), sarcomas-180 (70%), Pliss lymphosarcoses, M-I sarcomas, Walker’s carcinosarcomas (72–79%), and alveolar mucosal liver cancer PC-I (up to 89%), causes more than 80% inhibition of the growth of Pliss LPS, resistant to prospidin, and sarcoma 45, resistant to sarcolysin [4].

Compound 16 effectively affects the initial stages of the formation of metastases in the lungs, that is, at the moment of attachment of tumor cells to the lung tissue, and by its activity exceeds the effect of the approved drug Vinblastine by 2 times. At a dose of 100 μg/mL, it inhibited by 70–80% the growth of tumor cells from the ascitic fluid of patients with ovarian cancer.


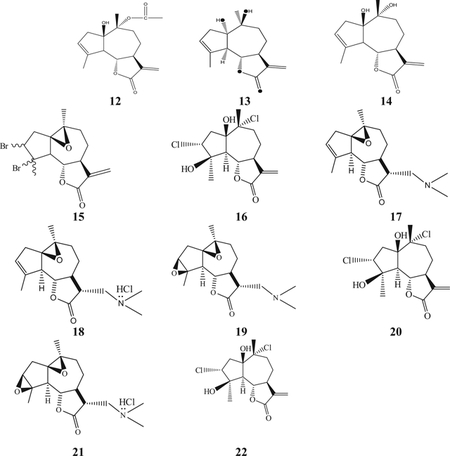


It has been proven that sesquiterpene lactones helenalin, its derivatives, parthenolide 3 modulate various cellular signaling pathways due to their high reactivity. Therefore, natural sesquiterpene lactones can be considered as specifically acting agents. On the other hand, due to the high reactivity of the thiol group in cysteine, as well as the amino group in lysine, they actively interact with the exomethylene group conjugated with the carbonyl of γ-lactone in the Michael type [21].

According to the results of the study of arglabin 6, it was determined that the drug has a pronounced cytotoxic effect against lung tumors and human ovarian cancer. At the same time, it does not have a depressant effect on hematopoiesis; it does not exhibit immunosuppressive activity, which distinguishes it favorably from the drugs currently used in the clinic, cyclophosphamide, and etoposide.

The sesquiterpene lactone parthenolide 3 inhibits nuclear factor kB (NF-kB), the STAT3 signaling protein, and NFAT-mediated transcription of anti-apoptotic genes. Thus, parthenolide 3 is a metabolic inhibitor to slow oncogenesis and inhibit tumor growth. Parthenolide 3 also inhibits gene expression induced by IL-6-type cytokines by blocking phosphorylation of the STAT3 signaling protein on tyrosine 705 (Tyr705), which explains its anti-inflammatory activity [22,23].

In addition to the antitumor effect of parthenolide 3, it has been found that this pseudoguayanolide inhibits 5-lipoxygenase and cyclooxygenase in leukocytes, as well as the release of 14C-serotonin from platelets and platelet aggregation induced by adrenaline, adenosine diphosphate, sodium arachidonate, and collagen ionophore [24].

A problem with sesquiterpene lactones, as with many other natural compounds in the field of chemotherapy, is their poor aqueous solubility and high toxicity, which often preclude their clinical use. One of the approaches to solving this problem is the synthesis of their water-soluble derivatives based on sesquiterpene lactones. Thus, using the example of thapsigargin 5, which induces apoptosis in prostate cancer cells, its interaction with a peptide carrier synthesized a water-soluble form of guayanolide, which is selectively activated by the protease of the specific antigen of metastatic prostate cancer. At the same time, a complete cessation of tumor growth was observed without significant toxicity in the prostate cancer xenograft model [25,26].

Overall, this “pre-patient research” approach presents a major challenge that can only be solved with an interdisciplinary approach from chemists, molecular biologists, pharmacists, pharmacologists, and clinicians.

Antitumor activity for arglabin 6 and its derivatives: dimethylamino arglabin hydrochloride 18, tetrachlorcarbenarglabin 23, and dimethylamino arglabin methyl iodide 24 was confirmed experimentally in *in vitro* tests on a culture of cancer cells NCI60 (Table 3).


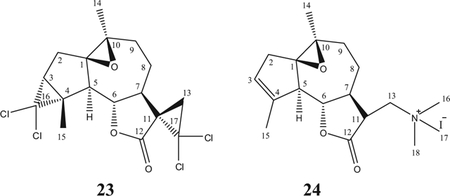


Based on the data in Table 4, it was found that arglabin 6, with a single injection into the culture of NCI60 tumor cells at a dose of 8.3 μM, exhibited antitumor activity, inhibiting the growth of the following cell lines:

- Inhibition of leukemia cells of the line: SR–97.8%; HL-60(TB) - 97.4%; K-562 – 73.8%;
- Colon cancer cells line: HCT-116–82.6%; HCT-15–75.4%; SW-620 – 70.9%; HT29 – 59.3%; COLO 205 - 57.6%;
- Melanoma cell line: UACC-62-74.2%; MDA-MB-435 - 65.8%; UACC-257 - 55.9%;
- Ovarian cancer cells line OVCAR-3-suppression of tumor cell growth by 69.6%;
- Kidney cancer cells of the line: TK-10-94.3%; CAKI-1-73.4%; RXF 393-67.3%;
- Prostate cancer cells of the DU-145 line by 88.5%;
- Breast cancer cell line: T-47D-73.5%; BT-549-72.1%; MCF7-71.1%.

At the same time, the average value of cell growth inhibition by the studied compound 6 for all 60 NCI60 human tumor cell lines is 45.9%.

When studying the culture of tumor cells NCI60 dimethylamino arglabin hydrochloride 18 with a single injection in dose 8.3μM, it was found that 18 inhibits the growth of cells of the following cell lines:

- Inhibits the growth of prostate cancer cells of the DU-145 line by 57.3%;
- Breast cancer cells of the MCF7 line-61.7%; T-47D - 58.9%.

On the culture of tumor cells NCI60 derivative of arglabin tetrachlorocarbene derivative 23 with a single injection at a dose of 8.3μM, exhibits antitumor activity, inhibiting the growth of RPMI-8226 tumor leukemia cells by 50.7%.

Dimethylamino arglabin methyl iodide 24 with a single injection at a dose of 8.3μM into a culture of NCI60 tumor cells inhibits the growth of the following cell lines:

- Leukemia cell line: RPMI-8226 – 91.5%; SR - 89.6%; HL-60(TB) - 80.3%; K-562 – 68.2%;
- Colon cancer cells line: HCT-116 – 81.8%; SW-620 – 73.4%; HCT-15 – 59.3%; HT29 – 54.9%;
- UACC-62 melanoma cells by 63.8%;
- Ovarian cancer cells of the OVCAR-3 line by 54.3%;
- Kidney cancer cells of the TK-10 line by 81.4%;
- Prostate cancer cells of the DU-145 line by 86.26%;
- Breast cancer cells of the T-47D line - 76.7%; MCF7 – 70.6%; BT-549 – 58.4%.

The model of glioblastoma in rats was experimental C6 glioma. In terms of morphology, the nature of invasive growth, and the pattern of expressed proteins, C6 glioma almost completely corresponds to human glioblastoma multiforme, which is the most invasive type of glioma, leading to human death within a year.

The antitumor activity of the arglabin derivative dimethylamino arglabin hydrochloride 18 was confirmed experimentally in *in vitro* tests on clonogenic rat C6 glioma cells, manifesting itself in the effective suppression of the proliferation of clonogenic cells.

It has been established that under conditions of administration of dimethylamino arglabin hydrochloride 18 at doses of 1 mg/ml (140 mg/m2 for humans) and 0.1 mg/ml (10 times lower than the therapeutic dose for humans), the proliferation of clonogenic C6 cells is significantly reduced by 17 and 5 times (2.95 ± 0.58% and 11.17 ± 2.7%, respectively) in relation to the control (51.47 ± 3.54%).

As the dose of dimethylamino arglabin hydrochloride 18 is reduced by several orders of magnitude (0.01 mg/mL, 0.001 mg/mL, and 0.0001 mg/mL), the proliferation of clonogenic cells is suppressed. When exposed to compound 18 at a dose of 2.5 mg/mL (300 mg/m^2^ for humans) on C6 clones, the proliferation of clonogenic cells significantly decreased by 2 times in relation to the control (4.19 ± 1.43% and 51, 47 ± 3.54%, respectively).

In this series of experiments, the effectiveness of suppressing the proliferation of clonogenic cells in C6 clones is observed with the injection of the studied compound 18 at doses of 1 mg/mL, 0.1 mg/mL, and 2.5 mg/mL, where a lethal dose (LD) was noted for clonogenic cells LD_94_, LD_79_, and LD_92_, respectively.

It was shown that dimethylamino arglabin hydrochloride 18 at a concentration of 20 mg/mL, 2 mg/mL, 0.2 mg/mL inhibits the proliferation of clonogenic cells by 10.4 times (LD_90_), 3 times (LD_67_), and 5 times (LD_80_) (4.96 ± 2.17%, 17.07 ± 2.06%, 10.3 ± 2.64%, respectively) compared with the control (51.47 ± 3.54%).

Thus, dimethylamino arglabin hydrochloride 18 at doses of 20; 2; 0.2 mg/mL, 1 mg/mL, 0.1 and 2.5 mg/mL effectively inhibited the proliferation of C6 clonogenic cells compared to controls in an *in vitro* experimental model of human glioblastoma multiforme. Probably, the pharmacological action 18 is associated with the inhibitory effect of farnesyl protein transferase.

### Cytotoxicity in the survival test of larvae of marine crustaceans A. salina (Leach).

For arglabin derivatives, their cytotoxicity was determined, which was evaluated in the survival test of larvae of marine crustaceans *A. salina (Leach)* under *in vitro* cultivation conditions (Brine shrimp toxicity bioassay method).

In the experiment, the effect of the studied substances at concentrations of 100 μg/ml, 10 μg/ml, and 1 μg/ml was studied on the survival rate of marine crustaceans *A. salina (Leach)* in Table 4.

It has been experimentally established that the presented samples exhibit cytotoxicity against the larvae of marine crustaceans *A. salina (Leach)*. At the same time, the IC50 of dimethylaminodioxyarglabin 10 is 104.2 μg/mL, while the IC50 of α-epoxyarglabin 7 and β-epoxyarglabin 8 are 89.4 and 92.9 μg/ml, respectively.

Compound 14 with an IC value of 64.2 μg/mL was the most active against the larvae of the marine crustaceans *A. salina (Leach)* (Table 4). Approximately the same effect was shown by compounds 12, 13, and 7. The IC50 values for these compounds are 77.8 μg/mL, 72.6 μg/mL, and 89.4, respectively (Table 4). IC50 compounds 8 and 10 are at and above the level of 100 μg/mL (Table 4), which indicates their low activity in this experiment relative to the reference drug.

Thus, samples of sesquiterpene lactones α-epoxyarglabin 7, β-epoxyarglabin 8, dimethylaminodioxyarglabin 10, hydroxyarglabin acetate 12, 1α,10β-dioxiarglabin 13, and 1β,10α-dioxiarglabin 14 exhibit cytotoxicity against larvae of marine crustaceans *A. salina (Leach)*.

## Conclusion

The conducted molecular docking shows that arglabin 6 and its derivatives predicted strong bonding on the receptors of DNA topoisomerase I, DNA topoisomerase II and farnesyl transferase, which suggests their destructive effect on the structure and functions of tumor cells, subsequently inducing their death, thereby exhibit cytotoxic and antitumor effects. Other anticancer drug targets cannot be ruled out. Molecular modeling data were supported by experimental studies.

According to the results of the experiments, it was found that the introduction of 6 atoms of bromine and chlorine into the molecule increases the antitumor activity of the synthesized samples 12, 13 against Pliss LPS, alveolar liver cancer RS-1, sarcoma M-1, and sarcoma 45.

Thus, the analysis of the results of computer modeling of the “ligand-target” complex and the pharmacological study of natural sesquiterpene lactones and their derivatives indicate the existence of a relationship between the chemical structure of their molecules, in particular, the presence of certain functional groups that can determine the final effect of molecules with the manifestation of the corresponding biological activity, in particular, cytotoxic and antitumor.

## Figures and Tables

**Table 1:** Binding energies of complexes of sesquiterpene lactone compounds with receptors of DNA topoisomerase I, DNA topoisomerase II, and farnesyl transferase

Compound	Receptor	Binding energy, kcal/mol
α-epoxyarglabin 7	DNA topoisomerase I	−6.0
	DNA topoisomerase II	−7.6
	Farnesyl transferase	−6.6
β-epoxyarglabin 8	DNA topoisomerase I	−6.8
	DNA topoisomerase II	−7.6
	Farnesyl transferase	−5.5
1 β,10α-dioxyarglabin 9	DNA topoisomerase I	−7.1
	DNA topoisomerase II	−9.0
	Farnesyl transferase	−7.0
Dimethylamino-1β,10α-dioxyarglabin 10	DNA topoisomerase I	−7.0
	DNA topoisomerase II	−7.8
	Farnesyl transferase	−7.3
Dimethylamino-1 β,10α-dioxyarglabin	DNA topoisomerase I	−8.4
hydrochloride 11	DNA topoisomerase II	−9.0
	Farnesyl transferase	−7.5

**Table 2:** Antitumor activity and toxicity of arglabin and its derivatives

Name of sesquiterpene lactone	Dose mg/kg	Growth inhibition of tumor strains, %																
		Lymphosarcoma Pliss	Vtelker’s carcinosarcoma	Guerin’s carcinoma	Sarcoma 45	Sarcoma M-1	Breast cancer RMK-1	Solid Ehrlich tumor	Alveolar liver cancer PC-1	Leukemia R-388	Leukemia L-1210	Resistant Options						
												Sarcomas 45				Lymphosarcoma Pliss		
												to 5-foruracil	to sarcolysin	to prospidin	to rubomycin	to rubomycin	to prospidin	to leucoefdin
1	2	3	4	5	6	7	8	9	10	11	12	13	14	15	16	17	18	19
Arglabin 6	30	57.6	41.1	48	23.0	55.6			32.1	43.0	34.0		59.7			44.1	31.0	
3,4β-epoxy-arglabin 8	30	72.1	36.4		88.8	78.4	59.6		70.4			66.0		70.4	78.6	78.0		79.8
Dimethylamino arglabin 10	50	56.0	30.0	85.1	79.0				42.0	80.1		52.1						
3,4-dibromarglabin 15	50	51.0	17.1	90.0	74.2				69.0	46.9		46.3						
Dimethylaminoarglabin hydrochloride 16	50	52.0	76.1	86.5	83.1				80.0	109.0		62.3						
Dimethylamino-3, 4β-epoxy-arglabin 19	50	64.6	43.1	31.4	58.1				38.0	51.0		11.2						
1β,4β-dioxy-3β,10α- dichlorarglabin 20	50	29.0	63.2	71.4	70.9				51.0	92.1		70.6						
1	2	3	4	5	6	7	8	9	10	11	12	13	14	15	16	17	18	19
Dimethylamino-3, 4β-epoxyarglabin	50	47.0	51.4	15.6	32.4				29.1	31.2		13.2						
hydrochloride 21																		
Chlorhydrinarglabin 22	50	49.1	38.4	43.1	21.0				31.0	20.4		15.2						
Koihamin	2	54.4	30.1		20.4							19.6						

**Table 3:** Antitumor activity of arglabin and its derivatives in the NCI60 human cancer cell culture model

Group/cell line	Arglabin 6	Dimethylamino Arglabin Hydrochloride 18	Tetrachlorocarbene -arglabin 23	Dimethylamino -arglabin methyl iodide 24
1	2	3	4	5
Growth of tumor cells, %				
Leukemia				
HL-60(TB)	2.63	98.28	103.12	19.66
K-562	26.23	83.71	87.93	31.81
MOLT-4	59.14	86.51	76.74	69.29
RPMI-8226	−5.73	60.46	49.35	8.51
SR	2.25	58.94	74.24	10.43
Non-small cell lung cancer				
A549/ATCC	83.94	99.60	93.42	103.60
HOP-62	67.33	85.20	97.97	58.41
NCI-H226	103.37	104.06	94.95	104.85
NCI-H23	62.36	102.45	96.42	78.22
NCI-H322M	114.06	110.90	96.02	105.72
NCI-H460	80.10	106.26	103.19	82.59
NCI-H522	−68.86	71.11	79.48	−68.41
Colon cancer				
COLO 205	42.37	101.68	110.34	54.14
HCC-2998	102.64	111.89	108.60	102.58
HCT-116	17.39	63.93	94.06	18.24
HCT-15	24.60	78.57	95.95	40.69
HT29	40.70	94.43	98.74	45.10
KM12	95.54	105.15	95.51	92.57
SW-620	29.05	61.20	109.32	26.60
T umors of the central nervous system				
SF-268	78.99	104.09	96.56	91.78
SF-295	89.81	104.37	78.88	
SF-539	54.28	99.36	89.12	61.68
SNB-19	101.01	100.34	82.07	100.58
SNB-75	62.66		59.07	80.73
U251	81.31	99.23	97.82	92.74
Melanoma				
MALME-3M	67.65	98.30	101.72	72.81
M14	67.86	96.66	99.15	78.16
MDA-MB-435	34.20	87.19	97.89	39.82
SK-MEL-2	84.20	108.75	111.65	86.38
SK-MEL-28	88.53	89.92	95.00	91.98
SK-MEL-5	76.31	94.99	89.68	83.87
UACC-257	44.07	80.63	108.75	61.32
UACC-62	25.79	85.96	72.39	36.21
Ovarian cancer				
IGROV1	98.42	114.40	94.88	103.08
1	2	3	4	5
OVCAR-3	30.37	102.61	106.26	45.67
OVCAR-4	86.04	97.49	90.07	101.83
OVCAR-5	82.60	104.46	88.42	76.68
OVCAR-8	72.01	99.89	99.21	98.38
NCI/ADR-RES	77.13	109.25	93.18	79.00
SK-OV-3	101.08	103.25	91.59	96.75
Kidney cancer				
786–0	73.74	100.79	95.25	82.38
A498	71.05	85.96	92.28	77.05
ACHN	53.47	88.70	100.42	59.54
CAKI-1	26.61	90.74	69.62	67.38
RXF 393	32.69	88.55	97.09	53.62
SN12C	64.30	114.38	96.51	74.15
TK-10	5.66	86.38	111.91	18.59
UO-31	81.56	100.55	77.41	87.22
Prostate cancer				
PC-3	56.38	82.27	82.86	71.67
DU-145	11.52	42.70	99.59	13.74
Mammary cancer				
MCF7	28.88	38.33	81.70	29.45
MDA-MB-231/ATCC	58.15	111.09	101.37	72.61
HS 578T	74.60	93.66	87.71	78,58
BT-549	27.88	63.40	86.00	41.64
T-47D	26.55	41.05	83.20	23.32
MDA-MB-468	−44.01	−22.99	82.29	−34.46
Mean tumor cell growth, %	54.15	89.30	91.96	61.54

**Table 4:** Cytotoxicity of arglabin derivatives in the survival test of larvae of marine crustaceans *Artemia salina (Leach)*

Substance	LD500, mg/mL
1	2
α-epoxyarglabin 7	89.4
β-epoxyarglabin 8	92.9
dimethylaminodioxyarglabin 10	104.2
hydroxyarglabin acetate 12	77.8
1α, 10β dioxyarglabin 13	72.6
1β,10α dioxyarglabin 14	64.2
1	2
Control: dimethyl sulfoxide	930.27
Reference drug: 13-dimethylamino-1,10β-epoxy-5,7α,6, 11 β(H)-guai-3,4-ene-6,12-olide hydrochloride	20.6
